# Cardiac Sarcoidosis Diagnostic Challenges and Management: A Case Report and Literature Review

**DOI:** 10.7759/cureus.24850

**Published:** 2022-05-09

**Authors:** Richa Jaiswal, Laseena Vaisyambath, Azadeh Khayyat, Nkechinyere Unachukwu, Bibimariyam Nasyrlaeva, Muhammad Asad, Stephanie P Fabara, Irina Balan, Sree Kolla, Rizwan Rabbani

**Affiliations:** 1 Internal Medicine, Medical University of South Carolina, Charleston, USA; 2 Pathology, Markham Stouffville Hospital, Markham, CAN; 3 Pathology and Laboratory Medicine, Brown University, Providence, USA; 4 Internal Medicine, Interfaith Medical Center, New York, USA; 5 Pathology and Laboratory Medicine, siParadigm Diagnostic Informatics, Pine Brook, USA; 6 Department of Pathology and Laboratory Medicine, Weill Cornell Medicine, New York-Presbyterian Hospital, New York, USA; 7 General Medicine, Universidad Catolica de Santiago de Guayaquil, Guayaquil, ECU; 8 Internal Medicine, Nicolae Testemițanu State University of Medicine and Pharmacy, Chisinau, MDA; 9 Biological Sciences, Staten Island Technical High School, Staten Island, USA; 10 Internal Medicine, Temple University Hospital, Philadelphia, USA

**Keywords:** case report, cardiac sudden death, arrhythmia, atrioventricular conduction abnormality, heart failure, cardiac manifestations, extrapulmonary sarcoidosis

## Abstract

Sarcoidosis can be presented as cardiac sarcoidosis (CS), which is challenging to diagnose due to its clinical silence. Ventricular arrhythmias and atrioventricular blocks can be fatal and cause sudden death in patients with cardiac sarcoidosis. Five percent of sarcoidosis patients have clinically evident cardiac sarcoidosis. However, autopsy reports and imaging studies have shown a higher prevalence of cardiac involvement. Early recognition is important to prevent such detrimental consequences. Cardiac sarcoidosis is increasingly being diagnosed owing to increased awareness among physicians and new diagnostic tools like MRI and positron emission tomography (PET) scan replacing traditional endomyocardial biopsy. A definitive diagnosis of CS remains challenging due to the non-specific clinical findings that can present similar symptoms of common cardiac disease; therefore, the imaging and biopsies are substantial for diagnosis confirmation. Pharmacological and Implantable devices are two main therapeutic approaches in cardiac sarcoidosis, in which steroids and pacemaker therapy have shown better outcomes. This review summarizes the available data related to the prevalence, prognosis, diagnosis, and management of cardiac sarcoidosis.

## Introduction

Cardiac sarcoidosis (CS) is diagnosed in almost one-third of patients with systemic sarcoidosis and is mostly diagnosed post-mortem by autopsy, as a very small percentage of patients are symptomatic [1]. The prevalence ranges from 10.9 per 100,000 for whites to 35.5 per 100,000 for blacks [2]. The natural course of CS is often unpredictable and can be aggressive at times if left undiagnosed and untreated [3]. CS is an uncommon but deadly manifestation of systemic sarcoidosis where the formation of noncaseating granulomas leads to inflammation of the pericardium, endocardium but most commonly, the myocardium. This progression from focal inflammation to scar formation in the left ventricular free wall to the papillary muscles results in the development of cardiomyopathies, arrhythmias, and even sudden cardiac death [4]. It may range from first-degree heart block to complete heart block, which accounts for 23%-30% of the patients with CS. Supraventricular arrhythmias are uncommon (15%-17%) and it is usually due to granulomatous formation at the sinus node. Progressive congestive heart failure accounts for 25%-75% of all cardiac-related deaths in patients with CS [3]. Having a high suspicion of CS when the patient presents with atrioventricular block or other symptoms is the mainstay of early diagnosis [1]. It is estimated that 5% of patients with systemic sarcoidosis develop CS, but up to 13%-25% of deaths in patients with systemic sarcoidosis have been attributed to CS in the USA [5]. 

Since there is no consensus for disease detection, monitoring, and treatment, diagnosis becomes a challenge when the only manifestation is cardiac dysfunction; however, it should be strongly suspected in patients with multi-systemic sarcoidosis [6]. Confirmed extra cardiac sarcoid with clinic suspicion (arrhythmias) and imaging studies like MRI are acceptable for probable sarcoid. Confirmed extra cardiac sarcoid with clinic suspicion (arrhythmias) and imaging studies like MRI are acceptable for probable sarcoid. Although endomyocardial biopsy is the gold standard, it is not mandatory [5]. This makes diagnosing isolated CS quite difficult, and 25% of CS cases present this way [7]. Patients with a strong probability or confirmed diagnosis for CS should be treated promptly to decrease the risk of fatal outcomes. Due to the high incidence of lung involvement most patients with sarcoidosis will present to a respiratory clinic, so pulmonologists play a key role in identifying and managing patients with cardiac sarcoidosis [8]. As mentioned above, a significant cause of mortality in these patients is arrhythmias and heart failure; therefore, optimizing detection methods is key to establishing disease, commencing treatment, and improving surveillance of disease progression. Considering these difficulties, we intended to bring this gap in the diagnostic criteria to light and highlight needed improvements in early detection and subsequent treatment. 

## Case presentation

A 40-year-old African American female with a history of hypertension presented to the clinic with seven days of dyspnea on exertion, headache, and dizziness. On examination, vitals were normal, chest X-ray showed cardiomegaly, bilateral hilar lymphadenopathy, and multiple lung nodules. Her electrocardiogram (ECG) showed a 2:1 atrioventricular (AV) block and right bundle branch block, followed by echocardiography which revealed right ventricular infiltration and reduction in systolic dysfunction. Based on these findings, differential diagnoses of mediastinal lymphoma, tuberculosis, and sarcoidosis were considered and evaluated.

The patient did not have a travel history. Cervical lymph node biopsy showed non-caseating granuloma formation, which confirmed the diagnosis of sarcoidosis. The patient was given corticosteroids 60 mg once daily, and a permanent pacemaker was implanted. At three months follow-ups, the patient's condition improved, and echocardiography findings subsided. Table 1 shows a summary of case reports.

**Table 1:** Shows the study name, author, year, study design, demographics, initial presentation, physical examination, investigations, complications/manifestations, management, and follow-up. ECG: electrocardiogram, QTc: corrected QT interval, LVEF: left ventricular ejection fraction, RVEF: right ventricular ejection fraction, CXR: chest X-ray, CHF: congestive heart failure, NYHA: New York Heart Association, RBBB: right bundle branch block, LV: left ventricular, ICD: international classification of diseases, CMR: calculated maximum response, SPECT: single-photon emission computerized tomography, PMCT: post-mortem computed tomography, PHT: pulmonary hypertension, BNP: brain natriuretic peptide, CTR: carpal tunnel release, LVEDP: left ventricular end-diastolic pressure.

Study Name, Author, Year	Demographics	Initial Presentation and Physical Examination	Investigations	Complications and Manifestations	Management	Follow-up
Tan J et al.; 2018	50 y/o male	Recurrent episodes of syncope for about 6 weeks BP 140/66 mmHg HR 54 bpm	ECG: sinus rhythm, first-degree atrioventricular (AV) block, prolonged QTc interval; Stress Test: pre-syncopal event nuclear stress test: mild reduced left ventricular ejection fraction (LVEF) 42%; Transthoracic echo: LVEF - 45–50%; CMR: focal transmural late Gd enhancement involving the basal anteroseptal wall, LVEF of 31%, and right ventricular (RV) EF 23%; CT Chest: mediastinal and bilateral hilar lymphadenopathy; Mediastinoscopy: non-caseating granulomas lymph node biopsies - cardiac sarcoidosis; Fungal infection and acid-fast bacilli staining - negative	Cardiac catheterization: LVEF 35-40%; complication - stent insertion required	A temporary pacemaker, dual antiplatelet therapy (Aspirin 81 mg daily and ticagrelor 90 mg twice daily), heart failure treatment (lisinopril 2.5 mg daily and metoprolol succinate 25 mg daily), and prednisone 60 mg orally three times a day with slow tapering.	One-year follow-up: LVEF 55% no recurrent syncopes
Ozaki et al.; 2009.	59 y/o female	Exertional dyspnea and general edema; known CHF NYHA Class III and Lung Sarcoidosis; BP: 142/90 mm Hg; HR 120 bpm, pretibial and facial edema I.	CXR: cardiomegaly; ECG: sinus tachycardia, first-degree atrioventricular block with complete RBBB; Echo: enlarged bi-ventricles, LV and RV end-diastolic dimensions 58 mm and 45 mm respectively, diffuse and severe hypokinesis of the LV with EF 25%; Echo with Doppler: severe tricuspid regurgitation (TR); Coronary angiogram: LV motion diffuse reduction, especially in the anteroseptal and apical areas; myocardial biopsy of the RV: septal epithelioid cell granuloma confirming sarcoidosis.	N/A	Furosemide, spironolactone, losartan, and beta-blocker therapy with pimobendane improved the CHF to NYHA class II on discharge.	One-year follow-up echo: increase in LVEF to 34%, shortening of LV end-diastolic dimension to 53 mm, RAP to 17 mmHg. Three years follow-ups: NYHA class I
Bhatti et al.; 2019	43 y/o male	History of lethargy, vomiting, anorexia, loose stools, and a day of palpitations. Past medical history of testicular cancer treated with right orchiectomy one year prior.	Sodium 121 mmol/L, potassium 6.4mmol/L; Echo: dilated LV cavity with moderately impaired LV systolic function (LVEF 40%–45%); CMR: LV impairment and arrhythmia.	N/A	Implantable cardioverter-defibrillator (ICD) for secondary prevention, a regimen of prednisolone	Asymptomatic, no ICD shocks in the last two years
Klunonaitis et al.;2017	34 y/o female	Palpitations	ECG: tachycardia 170 bpm, narrow QRS complex; Echo: LV dilatation with lateral and inferior walls motion abnormalities, LV apical aneurysm, LVEF 35%; CMR: similar findings to the echo.	Supraventricular arrhythmias	ICD for primary prevention of sudden cardiac death; immunosuppressive therapy with prednisone 60 mg.	N/A
Malli et.al.;2020	41 y/o male	Cough	CT Chest: enlarged hilar and mediastinal lymph nodes; bronchoalveolar lavage -lymphocytes 63%, CD4/CD8 ratio of 3.7; bronchial biopsy: non-caseating granulomas; cMRI: areas of late Gd enhancement consistent with cardiac sarcoidosis,	Worsening arrhythmias	Reduction in the number of ventricular arrhythmias (14,004) while on methylprednisolone 28 mg; pacemaker and ICD implanted while the patient was on atenolol.	Currently on methylprednisolone 8 mg; 2.5 years follow-up revealed a reduced (2790) number of ventricular arrhythmias.
Takamatsu et, al.2017	57 y/o female	Recurrent panic attacks induced by transient complete atrioventricular block. PE: stable and sustained ventricular tachycardia, that spontaneously returned to normal sinus rhythm.	ECG, Holter, labs, and CXR: no evident abnormalities.	Progressively worsening panic attack symptoms; two months after her initial visit - a complete atrioventricular block with 32s pause was revealed on a 24 hr Holter monitoring.	Cognitive behavioral therapy; pharmacotherapy - alprazolam 1.2 mg and mirtazapine 15 mg/day; permanent pacemaker; prednisolone 30 mg with an improvement of the cardiac function confirmed by the gallium scintigraphy.	19 months
Perez et, al.,2020	28 y/o male	Lacrimal duct obstruction	Preoperative ECG for lacrimal duct obstruction surgery: Q and T negative waves in inferior leads; Echo and CMR: LV aneurysm at basal segments of the inferior, posterior, and lateral walls with myocardial thinning and dyskinesia; CMR and CT Chest: bilateral nodular images in parotid glands, cervical, and thoracic lymphadenopathies; diagnosis confirmed by SPECT results and skin biopsy.	N/A	N/A	
Matsuda et, al.2017,	58 y/o female	Dyspnea on exertion (NYHA III); HR 50 bpm	Echo: LVEF 30%-35% with no asynergy of the LV, paroxysmal atrial fibrillation, complete left bundle branch block, or complete atrioventricular block.	Dizziness, dull chest, and ventricular tachycardia	ICD, angiotensin-converting enzyme inhibitor, beta-blocker, and warfarin.	Two years
Jotter and et al.2017.	32 y/o Caucasian female	Syncope episode two months prior to death; dizziness; sudden left deafness and left tinnitus eight years prior to this event.	PMCT examination: no abnormal findings; internal examination: signs of reanimation; the organs were normal at autopsy.	N/A	Prednisone 100 mg for five days. Four years later, recurrent deafness.	
Terasaki et, al,.2019	60 y/o male	Unconsciousness due to sick sinus syndrome; no therapy for sarcoidosis, recommended outpatient workup.	Echo: pericardial effusion, PHT with a transvalvular pressure gradient of 40 mmHg; 24-hour Holter: supraventricular extrasystoles with no additional abnormalities; CXR: cardiomegaly (CTR 55%) and pulmonary congestion.	Worsening dyspnea on exertion; PHT 50 mmHg and increased BNP 112.2 pg./mL	Prednisolone 30 mg daily, then maintenance dose tapered at 9 mg daily; catheter ablation for atrial arrhythmias; permanent pacemaker and pharmacotherapy consisting of prednisolone 10 mg, bisoprolol 1.25 mg and flecainide acetate 100 mg.	Four years
Chimezie U., et al	61 y/o African American male	Worsening dyspnea for two weeks; BP 180/80 mmHg	ECG: asymptomatic first-degree heart block and Mobitz type 1 AV block; Echo: LVEF 55%-60% with no regional wall motion abnormalities, increased posterior LV wall thickness 13 mm and septal 13 mm, RV volume overload.		Normal ECG after permanent pacemaker insertion.	Dyspnea and decreased ET with no pacemaker abnormalities; mildly elevated BNP 248 pg/mL, hypoalbuminemia, normocytic anemia; Echo: dilated RA, LVEF 30%-35% with diffuse hypokinesis, grade 2 diastolic dysfunction, elevated LVEDP; CT abdomen: liver mass, hepatomegaly and nodules in liver and spleen along with generalized lymphadenopathy.

## Discussion

Sarcoidosis is a granulomatous disease with multi-system involvement [9]; cardiac involvement can be found in 5% of cases of sarcoidosis [10]. In 27% of autopsy cases, cardiac sarcoidosis can be found [11], and CS is the second most common cause of death in sarcoidosis patients [12,13] prognosis and management of cardiac sarcoidosis have been challenging; the five-year survival of cardiac sarcoidosis was found 60%-70% [14,15]. Though in the absence of left ventricular dysfunction, the prognosis of cardiac sarcoidosis varies in 3% of cases with one-year mortality and 4%-100% of cases with 10-year [4,16].

Even though the clinical picture of cardiac sarcoidosis is highly variable, conduction abnormalities, arrhythmias, and heart failure are the most common presentation of cardiac sarcoidosis. Sometimes, cardiac sarcoidosis can be the only manifestation of sarcoidosis, and it should be suspected in young patients who are presenting with conduction abnormalities of unknown etiology [17]. Cardiac sarcoidosis usually occurs along with systemic disease; however, up to 25% of cases are isolated CS and tend to be clinically silent. They are usually nonspecific and occasionally fatal if symptoms are present, which can be diagnostically challenging [18]. These case reports have been summarized in Table 1.

The diagnosis of cardiac sarcoidosis can be confirmed by direct biopsy. The involvement of the heart is usually patchy, so the endomyocardial biopsy has been positive in only 20% of cases [19,20]. Almost 63% of cardiac sarcoidosis is isolated cardiac sarcoidosis that is confirmed histologically during transplantation or autopsy [7]. 

Moreover, patients may show ECG abnormalities ranging from nonspecific ECG finding to conduction abnormalities and ventricular arrhythmias, which cannot sometimes be found in 12-lead ECG without Holter monitoring [21] as in Tan J et al. case report ECG showed first-degree atrioventricular (AV) block and prolonged QT corrected for heart rate (QTc) interval while in Takamatsu et al. 2017 case report ECG did not show any abnormality. Although sudden death due to cardiac sarcoidosis is not rare, some infrequent manifestations of CS are often overlooked or not properly diagnosed at times [22-24]. Cardiac magnetic resonance (CMR) could provide suggestive imaging of cardiac sarcoidosis, including diffuse hypokinesis and left ventricle dilatation, thinning of the interventricular septum, or regional wall abnormalities with a possible right ventricular akinesis or aneurysm [25]. Although, positron emission tomography (PET-CT), a gold standard diagnostic method in CS that could be occasionally combined with a nuclear stress test, is recommended preferably for prognosis determination and treatment efficacy evaluation [25]. Despite the multitude of diagnostic methods for confirming the CS, their specificity and sensitivity remain limited [26]. Generally, the final diagnosis is established on clinicians’ opinions due to inadequate clinical trial validation [26]. The clinical and radiological findings and the beneficial treatment of the aforementioned case were strongly suggestive of CS; therefore, the care team did not proceed with an extensive workup.

Poor outcome is also associated with pulmonary hypertension secondary to extrinsic compression of pulmonary arteries by enlarged lymph nodes or cor pulmonale [27]. In the Terasaki et al. 2019 case report, the echo showed pericardial effusion pulmonary hypertension with a transvalvular pressure gradient of 40 mmHg. SCAD is likely still underdiagnosed due to misinterpreting results, but three cases have demonstrated the rarity of SCAD in CS [24]. Symptomatic pericardial involvement is rare, and few cases have been reported. The size of the effusion varies greatly, sometimes leading to a cardiac tamponade [28]. VT is also associated with heart failure and complete heart block that will eventually require an appropriate implantable cardioverter-defibrillator (ICD) [8]. The common first-line therapy is high-dose glucocorticoid; however, the response to immunosuppressive agents is unpredictable, and it is recommended that treatment includes implantable pacemaker or cardioverter defibrillation for primary prevention of sudden cardiac death [1]. ICD placement will prevent VT progression but will not prevent a recurrence. Therefore, concurrent treatment with anti-arrhythmic therapy is necessary for refractory VT [2]. 

The two main therapeutic approaches in CS patients are pharmacological management and invasive or device-oriented [29]. Steroid therapy and pacemaker therapy have shown better outcomes over the last 30 years. In our case report also, patients showed improvement after ICD transplantation and steroid therapy. ICD implantation and cardiac transplantation may also show improvements in management [30]. Even though the mainstay of treatment is immunosuppressants like corticosteroids, there are limited data on the optimal initiation, duration, and dosage. All the recommendations are based on small observational studies. 

## Conclusions

When it comes to understanding cardiac sarcoidosis, it looks like we have revealed the tip of the iceberg. Cardiac involvement is often asymptomatic in sarcoidosis cases, so early diagnosis and better screening protocol are essential for early cardiac sarcoidosis diagnosis and management. New imaging techniques have contributed to Global awareness of cardiac sarcoidosis. Capacity-building should be more focused on early detection along with the identification of signs which can be used to identify such cases of sarcoidosis.
